# An active learning approach to train a deep learning algorithm for tumor segmentation from brain MR images

**DOI:** 10.1186/s13244-023-01487-6

**Published:** 2023-08-25

**Authors:** Andrew S. Boehringer, Amirhossein Sanaat, Hossein Arabi, Habib Zaidi

**Affiliations:** 1 Division of Nuclear Medicine and Molecular Imaging, Geneva University Hospital, CH-1205 Geneva, Switzerland; 2 Geneva University Neurocenter, University of Geneva, CH-1211 Geneva, Switzerland; 3 Department of Nuclear Medicine and Molecular Imaging, University of Groningen, Groningen, Netherlands; 4 Department of Nuclear Medicine, University of Southern Denmark, Odense, Denmark

**Keywords:** MRI, Gliomas, Segmentation, Deep learning, Active learning

## Abstract

**Purpose:**

This study focuses on assessing the performance of active learning techniques to train a brain MRI glioma segmentation model.

**Methods:**

The publicly available training dataset provided for the 2021 RSNA-ASNR-MICCAI Brain Tumor Segmentation (BraTS) Challenge was used in this study, consisting of 1251 multi-institutional, multi-parametric MR images. Post-contrast T1, T2, and T2 FLAIR images as well as ground truth manual segmentation were used as input for the model. The data were split into a training set of 1151 cases and testing set of 100 cases, with the testing set remaining constant throughout. Deep convolutional neural network segmentation models were trained using the NiftyNet platform. To test the viability of active learning in training a segmentation model, an initial reference model was trained using all 1151 training cases followed by two additional models using only 575 cases and 100 cases. The resulting predicted segmentations of these two additional models on the remaining training cases were then addended to the training dataset for additional training.

**Results:**

It was demonstrated that an active learning approach for manual segmentation can lead to comparable model performance for segmentation of brain gliomas (0.906 reference Dice score vs 0.868 active learning Dice score) while only requiring manual annotation for 28.6% of the data.

**Conclusion:**

The active learning approach when applied to model training can drastically reduce the time and labor spent on preparation of ground truth training data.

**Critical relevance statement:**

Active learning concepts were applied to a deep learning-assisted segmentation of brain gliomas from MR images to assess their viability in reducing the required amount of manually annotated ground truth data in model training.

**Key points:**

• This study focuses on assessing the performance of active learning techniques to train a brain MRI glioma segmentation model.

• The active learning approach for manual segmentation can lead to comparable model performance for segmentation of brain gliomas.

• Active learning when applied to model training can drastically reduce the time and labor spent on preparation of ground truth training data.

**Graphical Abstract:**

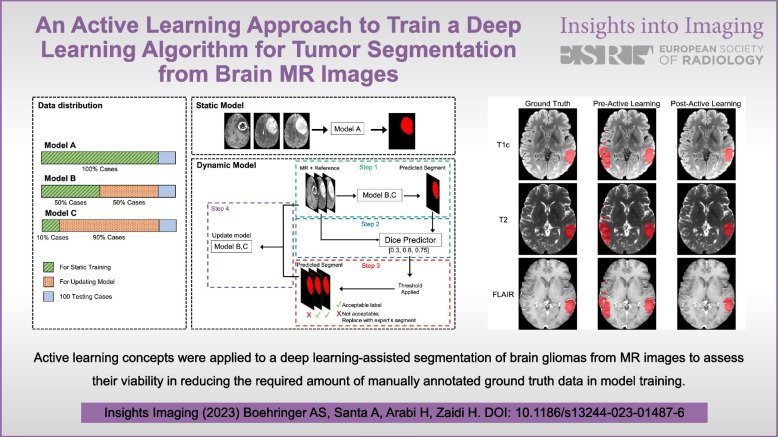

**Supplementary Information:**

The online version contains supplementary material available at 10.1186/s13244-023-01487-6.

## Introduction

With the substantial growth of deep learning-based approaches to solve complex problems, these techniques became naturally implemented in the medical image analysis field. The use of deep learning in medical imaging aids in both reducing the subjectivity of decisions by different experts as well as reducing the amount of time required for experts to spend on each case [1]. These benefits have the potential to provide major improvements in clinical diagnosis, treatment planning, and follow-up of individual patients [2].

While deep learning in medical image segmentation can be very useful, it also faces the issue of requiring large amounts of manually annotated data to serve as the ground truth reference during training. With segmentations for some tasks becoming more complex and requiring higher levels of accuracy with fewer errors, this ground truth need grows even further. Manually annotating ground truth data can be a huge burden, requiring the valuable labor effort and cost of trained experts. Manual segmentation of brain tumors, such as high-grade gliomas, for example, can take roughly 16 min per scan [3] and so in a dataset of around 1000 cases, the amount of time required just for preparing the manually segmented dataset can take hundreds of hours. Furthermore, although manual segmentation is considered as ground truth, a number of studies showed that it is not always accurate and may even include healthy tissues, in addition to being subject to inter- and intra-observer variability [4]. For some complex tasks, manually annotating enough data for training can become unfeasibly burdensome. For this reason, approaches to reduce the burden of acquiring adequate ground truth data for training deep learning algorithms is highly desirable.

To address this problem, analytical and deep learning-based methods have been proposed in the literature [5–9]. Using well-established augmentation methods, such as changing the images’ intensity, size, orientation, location, and skewness, are very common during model training and can improve the model performance. Robust-deep is also another augmentation method for brain images which can increase the dataset’s size and enhance the model’s robustness and performance by combining the different images from various patients in a realistic way [10]. Using deep learning-based augmentation methods has been shown to have a promising impact on models’ performance [11], but in the end, the performance of the model trained with these methods is limited and can lead to lack of robustness and reproducibility in new cases. For example, the deep learning-based models generate images which can be very similar to the initial images. Furthermore, one disadvantage of training the model with any type of augmentation model is the static learning procedure. Generally, the models are supposed to be trained, optimized, and used, all while the learning process can remain dynamic and never finish.

A potential approach toward reducing the ground truth burden is through the implementation of active learning techniques [12]. While often in machine learning the learner is a passive recipient of data to be processed, this “passive” role neglects the possibility of using feedback from the model to the learner’s benefit in an “active” role [13]. Active learning utilizes a semi-supervised approach in which the learner makes queries to influence which data is selected by the oracle for updating the model. When data is queried properly, it can drastically reduce the data requirements for some learning problems and greatly improve efficiency [14, 15]. In practice, active learning approaches are most beneficial in tasks that require very large datasets, often due to complexity, and have high cost and labor demands. The approach involves decreasing the amount of ground truth data needed for model training by first training the model with a smaller dataset, and then querying the unlabeled data for cases which would most benefit the model and adding them to the dataset. This approach makes use of the model’s prior knowledge to determine which instances would be most informative rather than random selection of instances to add. The approach adopted in this study follows a similar sampling strategy to the stream-based selective sampling strategy of active learning, in which unlabeled samples are queried and, if below a given threshold, added to the dataset in a semi-supervised manner [16]. In our study, this threshold was determined by the quality of the predicted segmentation, given by its Dice score, to demonstrate which cases are most challenging for the model that can be informative in further training. Cases below the threshold, with a low Dice score, would suggest that the segmentation model struggled with the segmentation and would require further training to learn how to properly segment this case and similar cases. Therefore, these lower quality cases would provide beneficial information to the model, whereas cases in which the model already performs well would not be as useful for further training as the model is already confident in its segmentation.

Active learning techniques have been applied to many medical-related challenges, including classification of sleep stages [17] or detecting seizures [18] from electroencephalogram (EEG), surgical workflow analysis [19], classifying cancer pathology reports [20], generating synthetic computed tomography (CT) images from MRI [21], and whole brain segmentation [22]. While the implementation of active learning techniques for deep learning in medicine is growing, the application of active learning in medical imaging and especially in deep learning-based segmentation is very sparse, with only a handful of studies. This study aimed to assess the application of an active learning approach to the development of a deep learning-based brain glioma segmentation model from MR images since applications of this method in this context are very sparse, with only a few of studies reported so far [23, 24].

## Materials and methods

### Dataset and preprocessing

The dataset used in this study was the publicly available training dataset provided for the 2021 RSNA-ASNR-MICCAI Brain Tumor Segmentation (BraTS) Challenge [2, 25, 26]. This dataset consisted of 1251 multi-institutional, multi-parametric MR images with pathologically confirmed glioma diagnosis. Each individual case contained pre-contrast T1-weighted MRI (T1), post-Gd contrast T1-weighted MRI (T1c), non-contrast T2-weighted MRI (T2), and non-contrast T2 Fluid Attenuated Inversion Recovery MRI (FLAIR), as well as a ground truth manual segmentations. Gliomas were divided into three image-based sub-regions for segmentation: Gd-enhancing tumor, necrotic core, and peritumoral edematous/invaded tissue. Example images can be seen in Supplemental Fig. 1.

For the purposes of this study, the three glioma segmentation labels were combined into a single label delineating the whole tumor (WT). While having a tumor segmented into its various histological sub-regions has more clinical relevance than a whole-tumor segmentation, the focus of this study was on assessing active learning concepts rather than developing a high-performance image segmentation model. Therefore, the more simplified whole-tumor segmentation allowed the focus to remain on active learning. With a reduced complexity of the segmentation model, less time and effort were needed for segmentation model training.

Additional preprocessing of the dataset was performed upon receival to prepare the dataset for implementation of machine learning algorithms. First, the images were cropped from 240 × 240 × 155 voxels to 160 × 216 × 128 voxels to remove excess blank space in the images using a maximum intensity projection (MIP) of the full dataset to determine cropping dimensions. Additionally, the images underwent bias field correction using the N4ITK algorithm [27]. The images were then normalized between 0 and 1 to avoid any intensity value biases. Normalization was done using the 98^th^ percentile value to reduce the impact of outlier voxels and prevent the intensity distributions from being skewed due to processing capacity of the NVIDIA Quadro K5000 GPU with 4 GB GDDR5 RAM (NVIDIA, Santa Clara, USA). The 1251 cases were split into a training set consisting of 1151 cases and a testing set, further referred to as T100, consisting of 100 cases. During training, 8% of the whole training data was randomly selected to be used as validation set.

### Active learning scheme

**Fig. 1:**
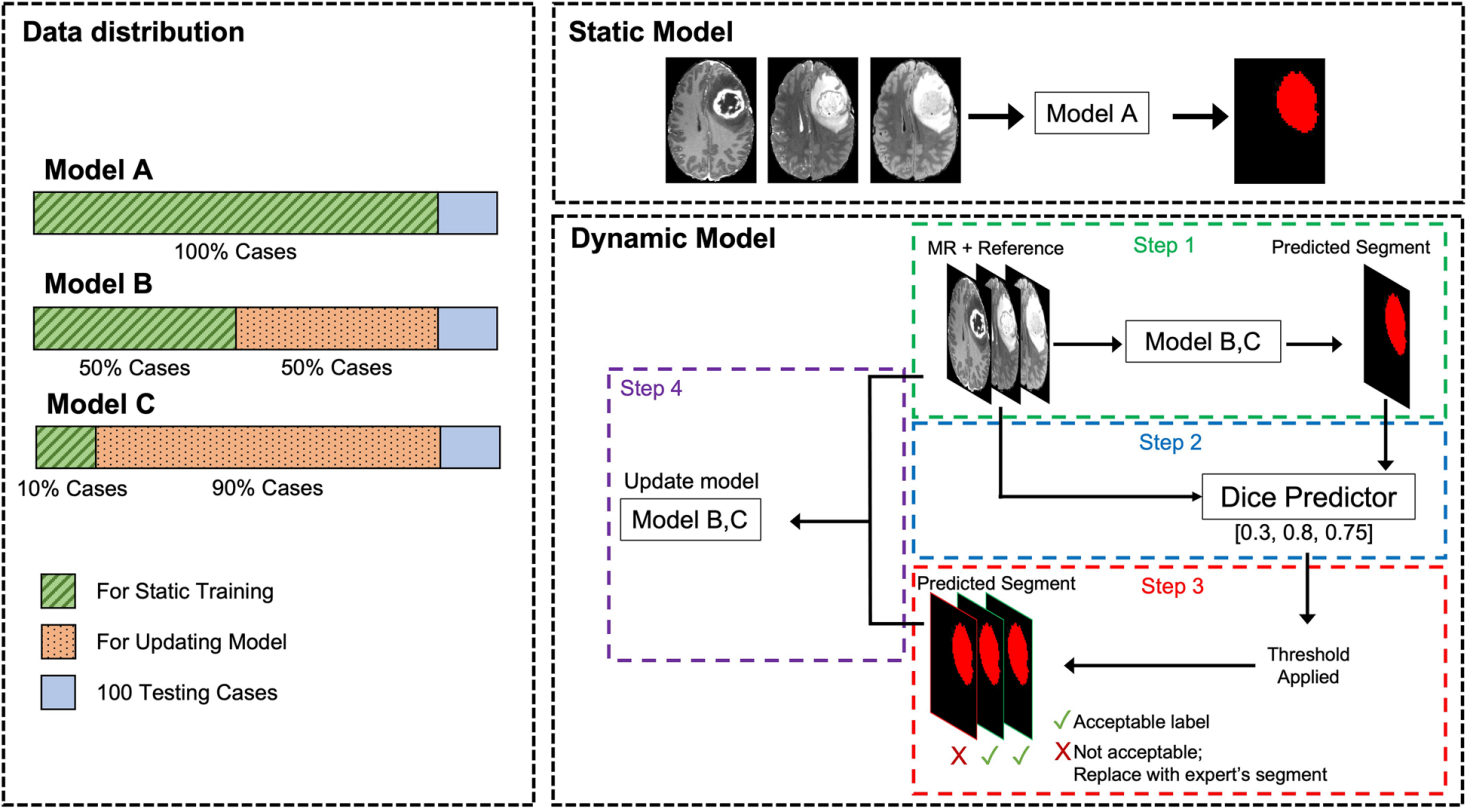
An example schematic demonstrating the active learning process

To assess the feasibility of active learning in medical image segmentation, three models were used. The first model served as a reference non-active learning model using all the data in the initial model training. The second model used only half of the dataset for initial model training with the other half set aside for use in active learning. The third model represented a severely reduced dataset and used only 100 cases in the initial training of the model, with the remaining set aside for active learning. After initial training with the two datasets of reduced size, a performance threshold was set and the performance of the model on the unseen active learning cases was computed for each case. The image sets passing through the threshold were then added to the training datasets, with predicted segmentations being added in place of the ground truth in cases which the models performed well in predicting segmentations. Model training then continued after this step and the results of the updated models were compared with the reference model. An example schematic demonstrating the active learning process can be seen in Fig. 1.

### Deep learning model

For the network architecture of the baseline reference model, the state-of-the-art, high-resolution, 3D convolutional HighRes3DNet [28, 29] network was used in the NiftyNet open-source platform [30]. HighRes3DNet was designed with the purpose of parcellating neuroanatomical structures from brain MRIs and is well suited to the task of this study. The network utilizes dilated convolutions and residual connections and contains 20 layers of convolutions [29]. To capture low-level image features, such as edges and corners, the first seven layers contain 3 × 3 × 3 voxel convolutions. The subsequent convolutional layers are first dilated by a factor of 2 then by a factor of 4 to capture mid-level and high-level image features [29]. Each convolutional layer is paired with a rectified linear unit (ReLU) layer and a batch normalization layer, and every two convolutional layers are grouped by residual connections. A final softmax layer provides classification scores for each voxel in the image. The architecture of the HighRes3DNet is provided in Supplemental Fig. 2.

### Implementation of active learning

A baseline model trained on all 1151 training cases (later referred to as “Model A”) was trained for 64 epochs to use as reference for comparison with models trained through active learning techniques. The training of the reference model utilized the HighRes3DNet described above with a 2D input, a batch size of 40, and initial learning rate of 0.01. The Adam optimizer was used as well as Dice loss with a decay of 1.0e − 5. The model took all three images (T1, T2, and FLAIR) jointly as input for each subject, as to provide maximal available information to the model and to not bias the model based on which sequence was provided for a given subject. In addition to the reference model trained on all 1151 training cases, two additional baseline models were trained: one using half of the training dataset (575 cases; Model B) and the other using only 100 training cases (Model C). Figure 1’s left panel shows the distribution of data for each model. These two models were trained using the same protocol and parameters as the reference model. Samples were partitioned into subsets through random selection.

For the implementation of active learning techniques after development of the three baseline models, the Dice score was used to evaluate which cases the model performed well with and which ones the model performed poorly with. For the two models with reduced training set size, Dice score was used to determine which additional cases would be beneficial to the training of the model and would need manual segmentation by an expert. With Model B, an iteration of the model shortly after the performance reached a plateau was selected for continuing with active learning. Using this model iteration, the resulting predicted segmentations were inferenced for the unused 576 training cases and the Dice scores for these predicted segmentations were computed. This earlier iteration was selected rather than the final model iteration so that any changes in model performance could be attributed to the adjustments of the training dataset rather than simply further training time. The resulting segmentations were then dichotomized based on their Dice scores into two categories: above or below a threshold score of 0.7. The threshold of 0.7 was selected from the literature on image validation suggesting Dice scores above 0.7 are considered to have a good overlap [31]. For those above a 0.7 Dice score, it was determined that the model did not struggle with the case and so the case was addended to the training dataset with the predicted segmentation in place of the ground truth. These cases represented those that would not require manual segmentation because the model had already learned how to adequately segment these images. For those below a 0.7 Dice score, it was determined that the model struggled with segmenting the image properly. These cases were addended to the training dataset but using the original ground truth segmentation and represented cases that required manual segmentation, as the model was still having trouble with the segmentation predictions. The process of replacing the predicted segmentation with the ground truth for these cases was equated to an expert manually segmenting/adjusting the case. Model training then continued with the updated dataset of a combined 575 initial training cases and 576 addended training cases for a total of 1151 training cases. The same procedure was followed for Model C using 200 additional training cases, constituting one round of active learning. For Model C, the process was repeated twice more with 600 additional training cases for an updated size of 900 training cases followed by 251 additional training cases to increase the size to the full dataset of 1151 training cases. The number of active learning cases in each step are provided in Supplemental Table 1.

In a real-world scenario, access to ground truth segmentations is limited and so computing Dice scores on unused training data is not feasible and any data with available ground truth data would be used in the training of the segmentation model itself. To address this issue, a secondary model was developed to predict the segmentation quality when provided with MR images and their predicted segmentation without the aid of the ground truth segmentation. The input to this model included T1c, T2, and FLAIR images as well as the segmentation probability map output of the segmentation model after the softmax layer. Rather than voxels being integer values depending on their predicted class of glioma or not glioma, the segmentation probability map takes the output from one step back when each voxel is represented by a value between 0 and 1 representing the probability of that voxel to belong to a given class, therefore providing extra information giving insight into the confidence of the model in its predicted segmentation. For this reason, the segmentation probability map was used in place of the predicted segmentation. Example input images can be seen in Supplemental Fig. 3. Because this secondary model is intended to inform on which segmentations performed by the model are acceptable or not, a researcher would require a threshold for which segmentations are either above or below the acceptable quality. To this end, a classification model is used as it allows the classification of segmentations into acceptable and unacceptable groups with defined thresholds.

To evaluate the effectiveness of the Dice score predictor when used for active learning decisions, the model’s results were compared with those of the initial “proof-of-concept” approach that incorporated the manual ground truth segmentations for determining Dice scores. To this end, the second active learning step of Model C was used containing 900 total training cases, with 100 initial training cases and 800 active learning cases. These 800 active learning cases did not include the 234 cases used in training of the classification model as to not contaminate the data with bias. To provide a comparison with the original Dice score method, the 800 active learning cases had the Dice scores of their predicted segmentations computed. Because the classification model used a threshold of 0.8 and above to signify an “Acceptable Quality” predicted segmentation, this threshold was used as a Dice cutoff with cases below 0.8 using the ground truth for training and cases above 0.8 using the predicted segmentation. The model using the original Dice method for comparison will be further referred to as Model D and the model using the results of the Dice score predictor as Model E. Both Models D and E then underwent training following the same procedure as the training for Models A, B, and C. Metrics of the resulting predicted segmentations were then computed and compared.

The subsections “Dice Score Prediction” and “Evaluation Strategy” are provided in Supplementary material.

## Results

Metric results for the “best performing” iteration of each of the three baseline models are summarized in Table 1, with the iteration selected through the Dice score due to this metric being used for thresholding further in the study. As expected, by reducing the training dataset size the models’ performance diminishes.

**Table 1:** Metric results for each baseline model

Model	Epoch	Sensitivity	Positive predictive value	Dice similarity coefficient	Jaccard similarity coefficient	Modified Hausdorff distance
Model A; 1151 training cases	52	0.912	0.906	0.906	0.834	3.309
Model B; 575 training cases	56	0.913	0.842	0.865	0.778	3.710
Model C; 100 training cases	57	0.849	0.825	0.825	0.718	4.317

Full metrics for these iterations can be seen in Table 2. Of the unseen dataset in Model B, 127 of the 576 cases (22.0%) were below the threshold of 0.7 Dice score and were replaced with the ground truth image. For the first round of active learning with Model C, 43 of the 200 unseen cases (21.5%) were below the 0.7 Dice score threshold. In the following rounds of active learning for Model C, 133 of the 600 unseen cases (22.2%) and 53 of the 251 unseen cases (21.1%) were below the threshold and replaced by ground truth segmentations for the 2^nd^ and 3^rd^ rounds of active learning, respectively.

**Table 2:** Metric results for each model iteration selected for the active learning process

Model	Active learning starting point	Sensitivity	Positive predictive value	Dice similarity coefficient	Jaccard similarity coefficient	Modified Hausdorff distance
Model B AL	23 epochs	0.872	0.859	0.854	0.757	4.045
Model C AL1	23 epochs	0.851	0.803	0.813	0.702	4.413
Model C AL2	38 epochs	0.869	0.815	0.830	0.725	4.215
Model C AL3	50 epochs	0.876	0.829	0.841	0.741	4.067

**Fig. 2:**
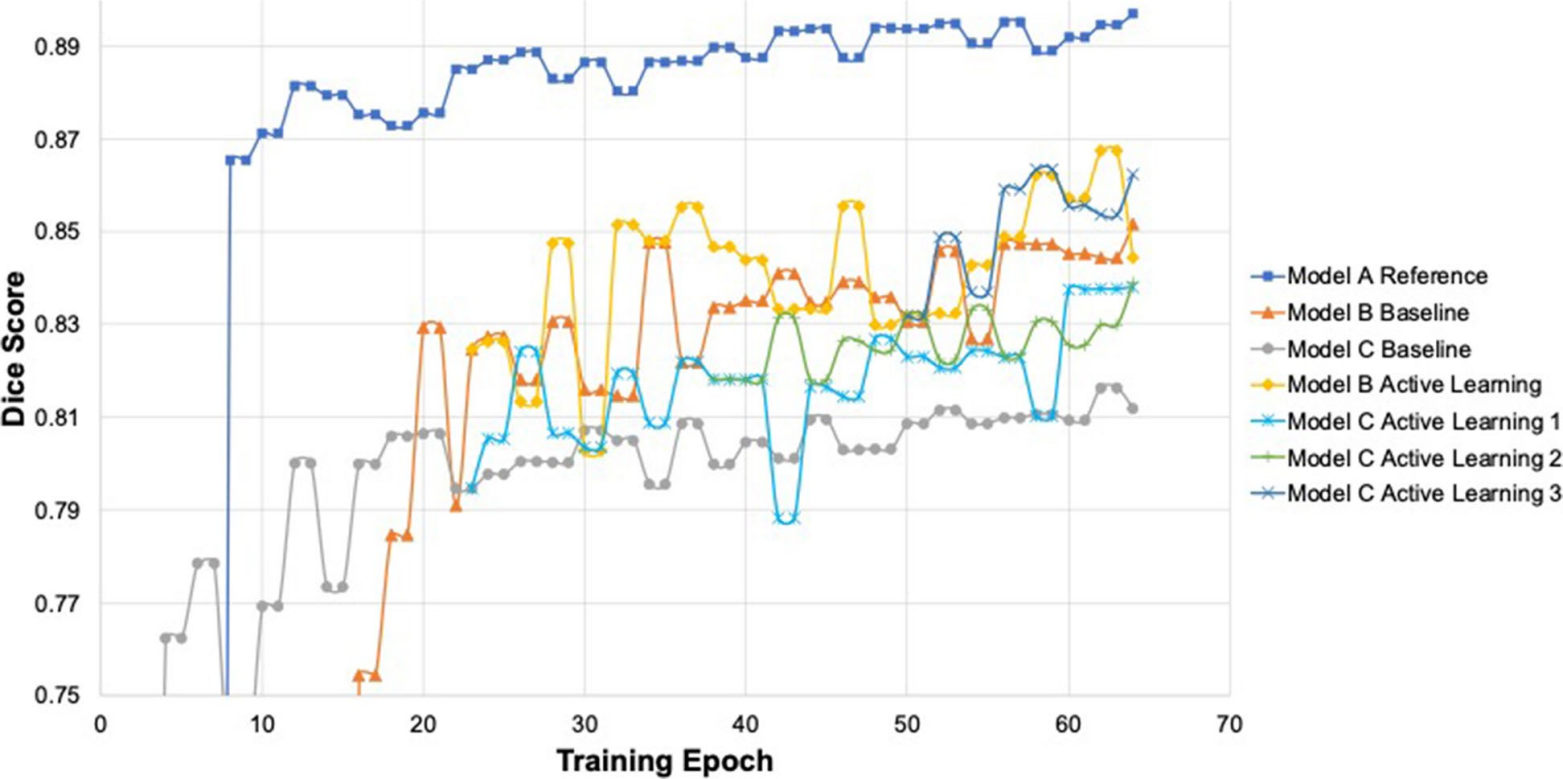
Training progress of reference Model A as well as baseline and active learning models for Models B and C

Figure 2 displays the training progress of each model’s Dice score. The three baseline models are shown in blue with squares (Model A), orange with triangles (Model B), and gray with circles (Model C). Model A shows the highest Dice score consistently through its training. Model B takes the longest for its Dice score to begin to plateau; however, the plateaued Dice scores are still above those of Model C. The active learning progress of Model B (yellow with diamonds) shows a higher peak Dice score than that of the baseline model. The same is seen for Model C in which each round of active learning for Model C shows improvement. The baseline has the lowest peak Dice score, followed by the first round of active learning (light blue with asterisks), the second round of active learning (green with vertical lines), and finally the third round of active learning (navy with x’s) with the highest Dice scores.

Table 3 includes the reference Model A, the baselines of Models B and C, and the final post-active learning results for Models B and C. In terms of peak average Dice scores, the post-active learning models of Models B and C both showed improvement, with the overlap between the predicted and ground truth segmentations increasing an average of 0.5% for Model B and 4.3% for Model C. Though the improvements for Model B were not as pronounced, Model C’s improvements from pre- to post-active learning were more notable. Additionally, through active learning Model C’s sensitivity improved from 0.849 to 0.907 and PPV from 0.825 to 0.845. The intersection between the predicted and ground truth segmentations represented by the Jaccard Similarity Coefficient also increased through active learning from 0.718 to 0.777, and the Modified Hausdorff Distance showing the distance between the two sets of voxels from the predicted and ground truth segmentations improved with a decrease of 12.9%.

**Table 3:** Metric results of the pre-active learning and post-active learning segmentation models

Model	Epoch	Sensitivity	Positive predictive value	Dice similarity coefficient	Jaccard similarity coefficient	Modified Hausdorff distance
Model A; Reference	52	0.912	0.906	0.906	0.834	3.309
Model B; Baseline	56	0.913	0.842	0.865	0.778	3.710
Model B; Post-active learning	63	0.882	0.876	0.870	0.780	3.837
Model C; Baseline	57	0.849	0.825	0.825	0.718	4.317
Model C; Post-active learning	57	0.907	0.845	0.868	0.777	3.761

**Fig. 3:**
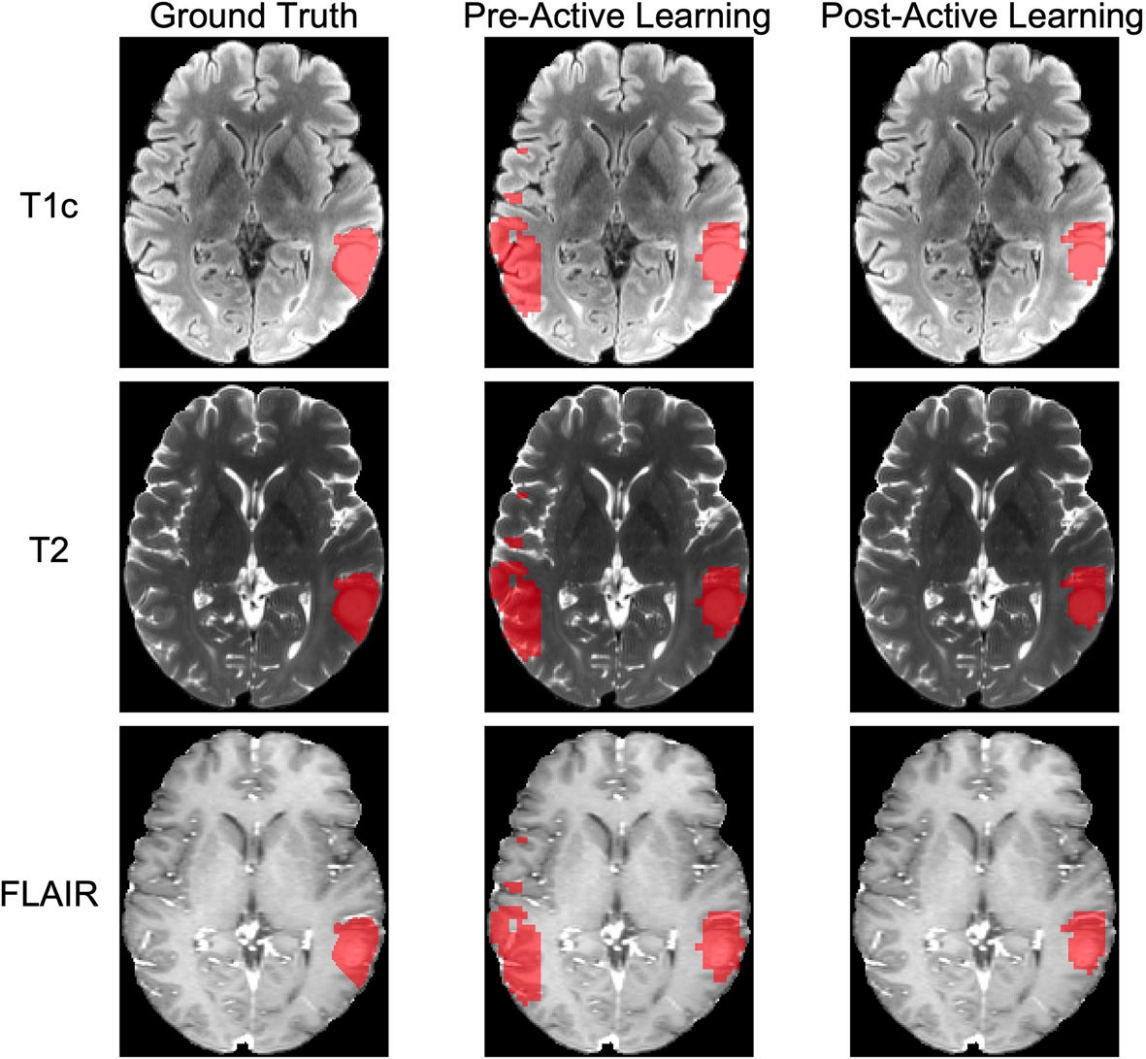
Ground truth, pre-, and post-active learning predicted segmentations for T1c, T2, and FLAIR images for a representative case in Model C

**Fig. 4:**
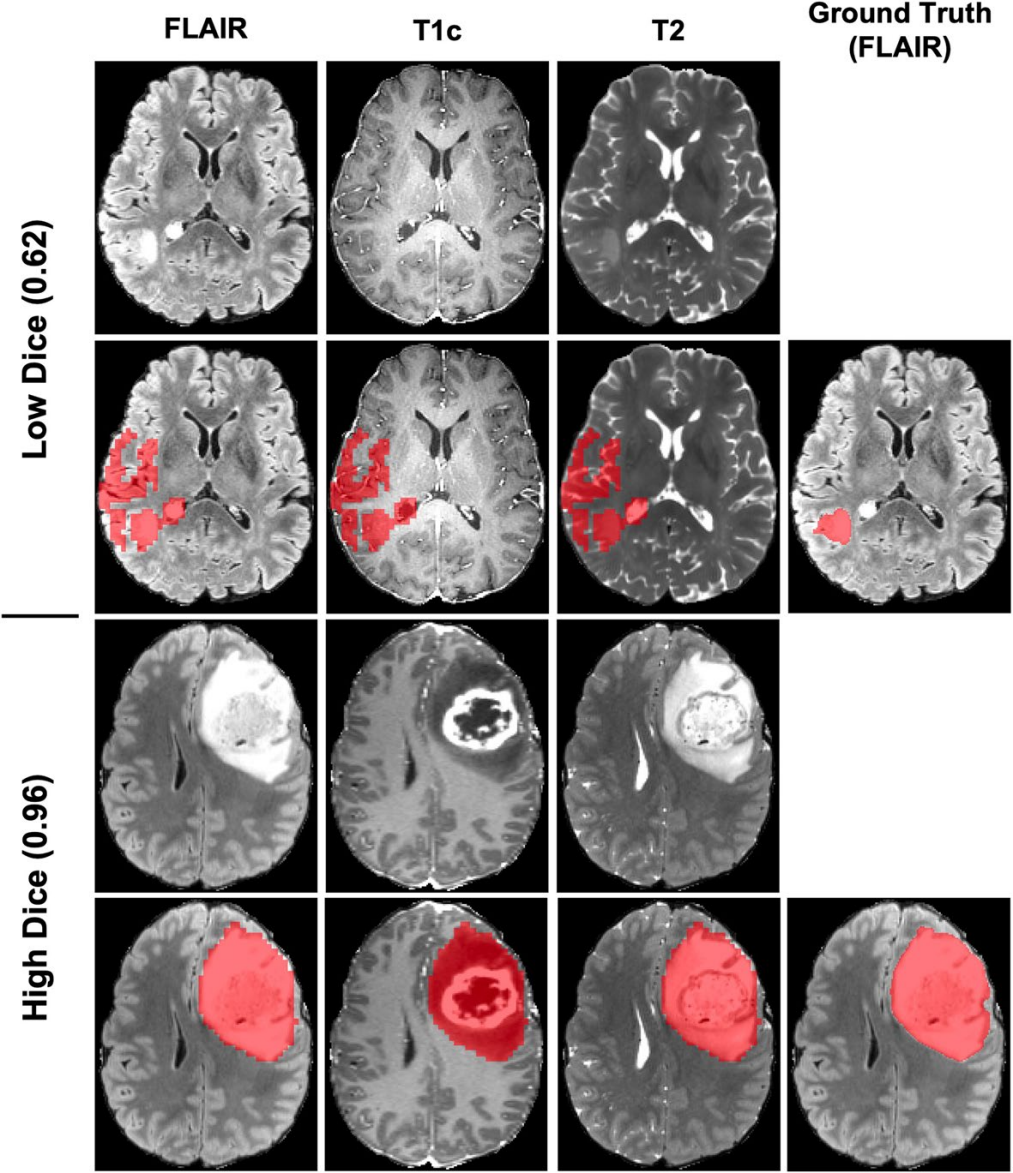
Example cases from Model C with low (above) and high (below) Dice scores and their predicted and ground truth segmentations for T1c, T2, and FLAIR

For qualitative visual assessment of segmentation prediction, a representative example case of segmentations before and after active learning from Model C can be seen in Fig. 3 for the three MR sequences. In the example case, before active learning the model predicted a large region opposite the glioma to be glioma tissue. Visually, the incorrectly segmented region appears larger than the ground truth segmentation. After active learning, this incorrectly segmented region has disappeared and only the true glioma has been segmented. For additional qualitative assessment, two example cases from Model C after the active learning process are displayed in Fig. 4 representing low Dice score and high Dice score cases. In the high Dice score case with an individual case Dice score of 0.96, the glioma is very prominent in the MR images both in terms of size and contrast with the background brain tissue. In the low Dice score case with an individual case Dice score of 0.62 on the other hand, the glioma is much smaller and less prominent in the MR images. The predicted segmentation by the model over-segments the glioma, capturing healthy brain tissue in the segmented volume.

**Fig. 5:**
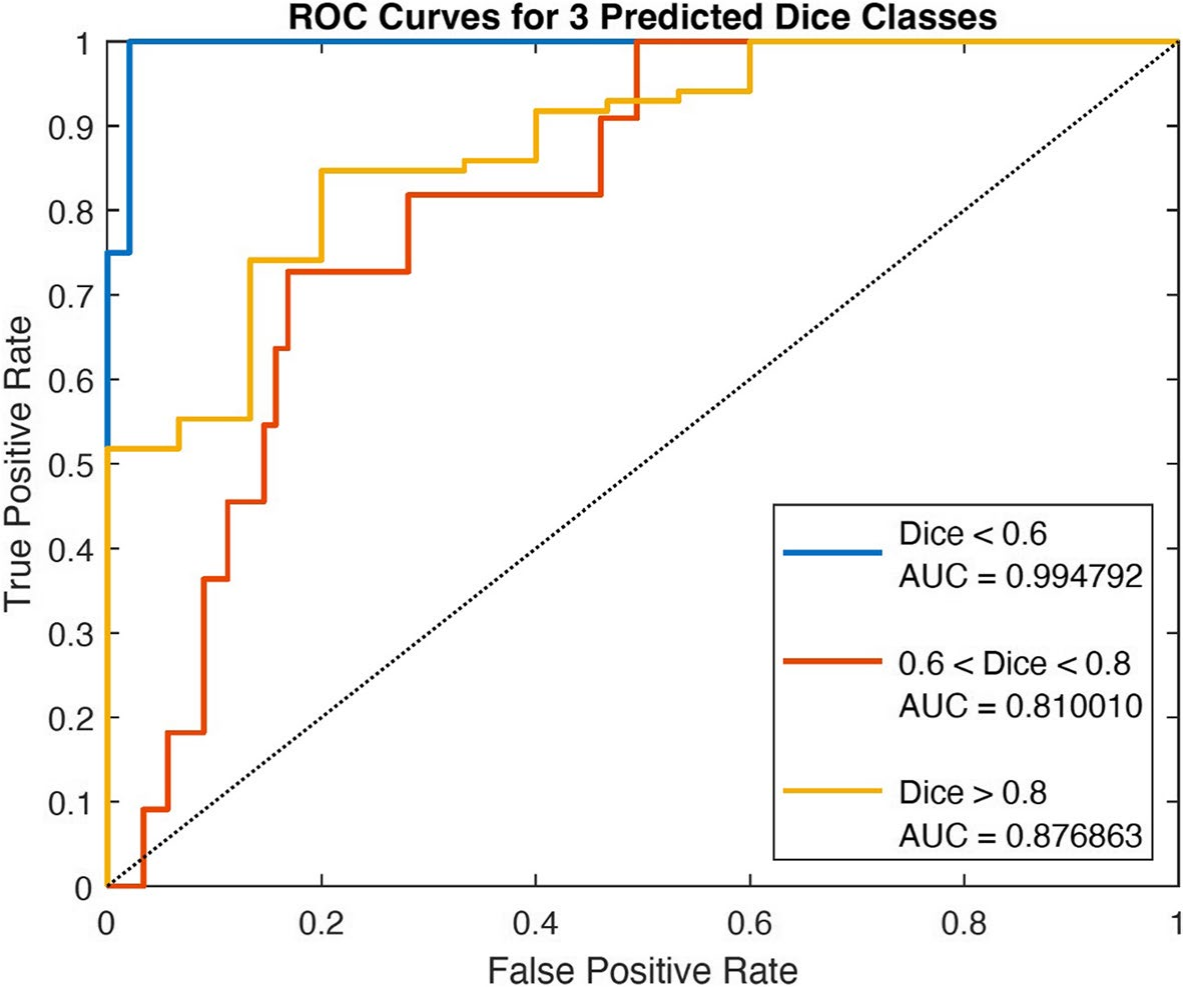
ROC curves for the three classes predicted by the Dice score predictor for predicting the quality of segmentation

A confusion matrix of the model to classify predicted segmentations can be seen in Supplementary Fig. 4 for the classes “Poor Quality” in which a physician needs to segment the image from scratch, “Acceptable with Adjustments” in which a physician needs to check and edit the segmentation, and “Acceptable Quality” in which the segmentation does not require any checking or editing by a physician. Of 100 total T100 cases, 82 were classified to the correct class while 18 were misclassified for an accuracy of 82%. Of the four cases in the “Poor Quality” class, three (75%) were classified correctly with the incorrectly classified case assigned to the “Acceptable with Adjustments” class. For the 11 cases in the “Acceptable with Adjustments” class, 6 (54.5%) were classified correctly while 5 were incorrectly classified to the “Acceptable Quality” class. Of the 85 cases in the “Acceptable Quality” class, 73 (85.9%) were classified correctly while 12 were incorrectly classified to the “Acceptable with Adjustments” class. Of the 18 misclassified cases, 13 (72.2%) were misclassified into a class that would still require assessment by an expert (“Poor Quality” or “Acceptable with Adjustments”). Additional metrics of the classification model results can be seen in Table 4. Figure 5 displays the ROC curves for the three predicted Dice score classes with AUC scores listed. The “Poor Quality” class of Dice scores less than 0.6 showed the highest AUC of 0.995, followed by the “Acceptable Quality” class of Dice scores above 0.8 with an AUC of 0.877, and finally the “Acceptable with Adjustments” class of Dice scores between 0.6 and 0.8 with an AUC of 0.810.

**Table 4:** Metric results for each class of the classification model

Class	Sensitivity	Specificity	Positive predictive value	*F*-score	AUC
Poor Quality (4 cases)	0.750	1.000	1.000	0.857	0.995
Acceptable with Adjustments (11 cases)	0.545	0.938	0.316	0.400	0.810
Acceptable Quality (85 cases)	0.859	0.455	0.936	0.896	0.877

**Fig. 6:**
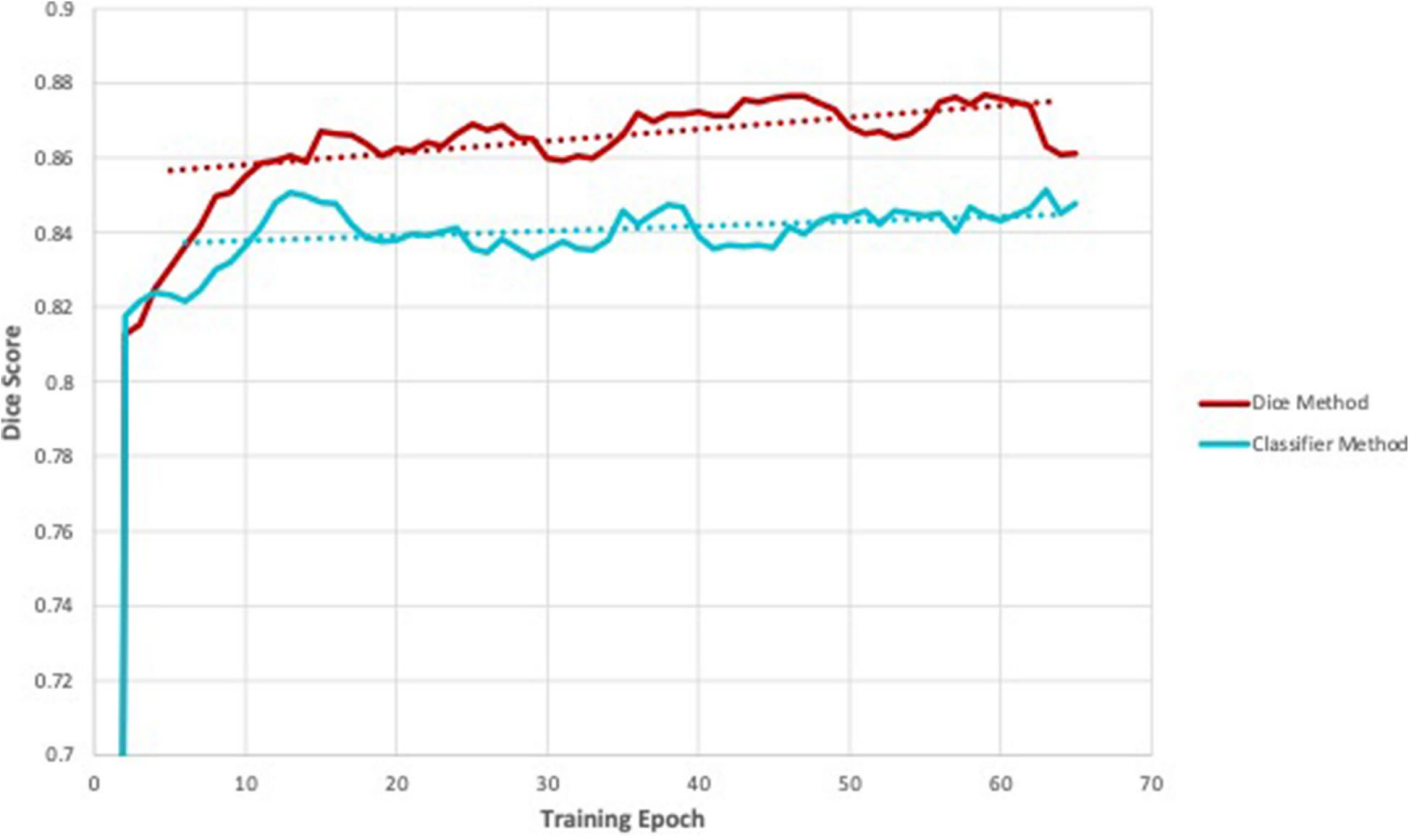
Training progress of the segmentation models used to evaluate the effectiveness of the classification model

Among the 800 active learning cases for Model D using a threshold of 0.8 Dice score, 486 (60.75%) had a Dice score above 0.8 and only required the predicted segmentations for further model training. Of the 900 total training cases for this step, Model D used 414 manual ground truth segmentations (46.0%). For Model E using the classification model’s predicted classes, 512 of the 800 active learning cases (64.0%) were in the “Acceptable Quality” class and only required the predicted segmentations for further model training. Of the 900 total training cases, only 388 required manual ground truth segmentation (43.11%). Metric results for the post-training performance of Models D and E can be seen in Table 5. Dice score using the classification model in the absence of ground truth data decreased by a Dice score of 0.025 compared to the model utilizing ground truth data to make decisions. Following this trend, the other metrics were slightly better for Model D though not by much. A graph of the training progress for Models D and E can be seen in Fig. 6.

**Table 5:** Metric results for the reference Dice score method Model D and the classification model method Model E

Model	Epoch	Sensitivity	Positive predictive value	Dice similarity coefficient	Jaccard similarity coefficient	Modified Hausdorff distance
Model D; Dice score method	51	0.918	0.864	0.885	0.802	3.563
Model E; Classification model method	60	0.904	0.835	0.860	0.764	3.899

## Discussion

The classification model was highly sensitive to cases with “Poor Quality” predicted segmentations of low Dice score (< 0.6) which can be very useful in identifying the problematic cases. Additionally, though there were 18% of cases misclassified, many of these would not negatively impact the results. For example, though there were 12 cases of “Acceptable Quality” misclassified to be in the class for “Acceptable with Adjustments”, this incorrect class would simply suggest that an expert would need to make minor adjustments and in doing so the expert would see that the quality of the segmentation is acceptable. Therefore, being misclassified into a lower class does not negatively impact the results. There were only 6 of the 100 T100 cases misclassified into a class of better quality, with one of these being misclassified from “Poor Quality” to “Acceptable with Adjustments” which would still warrant an expert to visually assess the segmentation. Additionally, none of the misclassifications jumped between “Poor Quality” and “Acceptable Quality” and only into “Acceptable with Adjustments”. When taking these instances into account, 95% of the cases were either classified correctly or if incorrectly then into a class that would still require an expert to visually assess the predicted segmentation and adjust if necessary.

Overall, the active learning approach adopted in this study demonstrated substantial reductions in the required amount of manually segmented ground truth data for model training. Despite these results, however, the approach faces several limitations. First, this study primarily focused on the Dice similarity coefficient for assessment of the segmentation quality. This led to a one-dimensional decision on the segmentation quality rather than incorporating multiple metrics into the decision, such as sensitivity, positive predictive value, Jaccard similarity coefficient, and modified Hausdorff distance. Second, due to the nature of metrics like Dice similarity coefficient in which it compares the predicted segmentation with the ground truth segmentation, a secondary model was required for a “real-world” scenario in which the ground truth data is not already present. This second step introduces further potential for error and so having the process condensed to a single step would be ideal. Lastly, the images were resampled to a smaller resolution and the initial 3-label segmentation was combined to a single-label segmentation to reduce complexity. While this greatly affected the quality and usability of the final predicted segmentations from the model, the overall accuracy and strength of the segmentation result was not important in this study but rather the viability and benefit of active learning concepts in model development. Despite these limitations, this study demonstrated that active learning can greatly reduce the efforts of preparing ground truth data for training segmentation models. With the querying technique being a crucial aspect in the success of the active learning model [18, 32], future studies can explore alternate techniques and potentially improve results even further. While the accuracy of the segmentation model used in this task does not yet meet the standards for clinical use, this study serves as a baseline for future work to further delve into the application of active learning for medical image segmentation, perhaps testing other active learning techniques as well.

## Conclusion

In this study, active learning concepts were applied to a deep learning segmentation of brain gliomas from MR images to assess their viability in reducing the required amount of manually annotated ground truth data in model training. It was demonstrated that using this active learning approach, more than 60% of the dataset did not require manual segmentation for adequate training of the model, suggesting that active learning when applied to model training can drastically reduce the time and labor spent on preparation of ground truth training data. Furthermore, a secondary model was developed to classify the predicted segmentations into three classes based on their quality. The results of the classifier suggested that in addition to active learning concepts being greatly beneficial toward streamlining model training for medical image segmentation tasks, approaches that do not require prior knowledge of the unseen data are feasible as well.

### Supplementary Information

**Additional file 1: Supplemental Table 1.**Summary of the number of training cases used in each active learning model. **Supplemental Figure 1.** A representative case demonstrating (top panel) representative images above from left to right: T1c, T2, FLAIR, and manual ground truth segmentation (red: necrotic core (NCR), blue: Gd-enhancing tumor (ET), green: edematous/invaded tissue (ED)). Bottom panel demonstrates the union of the three segmentation labels for NCR, ET, and ED into a single segmentation label of the whole tumor (WT). **Supplemental Figure 2.** Network architecture of the Dice score predictor model for classifying predicted segmentation quality. **Supplemental Figure 3.** Example input images for the Dice score predictor including from left to right: T1c, T2, FLAIR, and the predicted segmentation probability map. **Supplementary Figure 4.** Confusion matrix for the classification of predicted segmentations into “Poor Quality”, “Acceptable with Adjustments”, and “Acceptable Quality”.

## Data Availability

The data sets that support the findings of this study are publicly available. The code developed will be available on Github upon publication of this work.

## Abbreviations

ASNR: American Society of Neuroradiology
BraTS: Brain Tumor Segmentation
CT: Computed tomography
FLAIR: Fluid Attenuated Inversion Recovery
MICCAI: International Conference on Medical Image Computing and Computer Assisted Intervention
MIP: Maximum intensity projection
MRI: Magnetic resonance imaging
PPV: Positive predictive value
ReLU: Rectified linear unit
RSNA: Radiological Society of North America
SGDM: Stochastic gradient descent with momentum
WT: Whole tumor
