# Supplementary material for: An active learning approach to train a deep learning algorithm for tumor segmentation from brain MR images
Source: Insights Imaging. 2023 Aug 25;14:141. doi: 10.1186/s13244-023-01487-6 (PMC10449747; doi:10.1186/s13244-023-01487-6)
Supplement: Supplementary file 1 — Additional file 1: Supplemental Table 1.Summary of the number of training cases used in each active learning model. Supplemental Figure 1. A representative case demonstrating (top panel) representative images above from left to right: T1c, T2, FLAIR, and manual ground truth segmentation (red: necrotic core (NCR), blue: Gd-enhancing tumor (ET), green: edematous/invaded tissue (ED)). Bottom panel demonstrates the union of the three segmentation labels for NCR, ET, and ED into a single segmentation label of the whole tumor (WT). Supplemental Figure 2. Network architecture of the Dice score predictor model for classifying predicted segmentation quality. Supplemental Figure 3. Example input images for the Dice score predictor including from left to right: T1c, T2, FLAIR, and the predicted segmentation probability map. Supplementary Figure 4. Confusion matrix for the classification of predicted segmentations into “Poor Quality”, “Acceptable with Adjustments”, and “Acceptable Quality”. [file 13244_2023_1487_MOESM1_ESM.pdf]

Supplemental Material

A. Dice Score Predictor

The Dice scores were separated into three classes: "Poor Quality" for those below 0.6 Dice score, "Acceptable with Adjustments" for those between 0.6 and 0.8 Dice score, and "Acceptable Quality" for those above 0.8 Dice score. The 0.8 Dice score threshold was selected as the average Dice score of all models submitted to the 2021 BraTS Challenge, whereas the 0.6 Dice score was selected as being slightly above the central 0.5 Dice score. A predicted segmentation in the "Poor Quality" class would need to be segmented completely manually by an expert, a segmentation in the "Acceptable with Adjustments" class would need some minor adjustments by an expert before being accepted, and a segmentation in the "Acceptable Quality" class could be accepted with possibly only a brief visual check by an expert. The predicted segmentations and Dice scores were taken from the iteration of Model B used for active learning. The unused 576 training cases were used for training of the Dice score predictor as to not use cases that the initial model was trained on. Additionally, due to the performance of the initial segmentation model, the Dice scores of the training set were skewed towards higher Dice scores. To counter this, the amount of training cases for each class was balanced, resulting in 78 cases per class for a total of 234 training cases. For consistency, the testing set used for this classification model was the same T100 set used for testing the initial segmentation models.

For the Dice score predictor, a model was trained in Matlab R2022a Deep Learning Toolbox (MathWorks, Portola Valley, United States) for 60 epochs with an initial learning rate of 0.1 using a piecewise schedule that dropped the learning rate by a factor of 0.1 every 10 epochs. The network consisted of 61 total layers, beginning with a 3D input layer followed by 14 blocks consisting of a max pooling layer, convolution layer, batch normalization layer, and ReLU layer. The first of these blocks did not have a max pooling layer. For the first 7 blocks, the subsequent convolutions were dilated by a factor of 2 and for the remaining blocks they were contracted by a factor of 2. Following these blocks, the network ended with a dropout layer with a value of 0.1, two fully connected layers of size 512 and 3, respectively, and a softmax layer before the final classification layer. A batch size of 64 was used during training with an initial learning rate of 0.1 and momentum of 0.9. Stochastic gradient descent with momentum (SGDM) was used as the optimizer along with 5.0e-5 L2 regularization and a 0.5 gradient threshold.

B. Evaluation strategy

After training of the baseline models, predicted segmentations of T100 were inferenced for each model. From these predicted segmentations and the ground truth segmentations, various evaluation metrics could be calculated to determine the agreement between the predicted and ground truth segmentations. These metrics included Sensitivity, Positive Predictive Value (PPV), Dice Similarity Coefficient (further referred to as Dice score), Jaccard Similarity Coefficient, and Modified Hausdorff Distance. For final model selection, the average Dice score of the T100 cases

was computed for each iteration in each model. The "best" iteration for a given model was determined as the iteration with the maximum average Dice score and from here the rest of the metrics of this iteration were reported.

C. Quantitative evaluation

Each voxel in the image can then be classified as either true positive (TP), true negative (TN), false positive (FP), or false negative (FN). The sensitivity is defined as the ratio of TP to the combined TP and FN and can be seen in equation 1. In other words, sensitivity describes the ability of the model to correctly identify voxels belonging to the glioma. Sensitivity is often accompanied by specificity, however because specificity is dependent on the volume of the glioma, which varies greatly from patient to patient, it does not convey any useful information [1]. An alternative to specificity that can be used is PPV, which is defined as the ratio of TP to the combined TP and FP seen in equation 2. This describes the proportion of correctly identified glioma voxels.

$$Sensitivity = \frac{|TP|}{|TP| + |FN|} \tag{1}$$

$$PPV = \frac{|TP|}{|TP| + |FP|} \tag{2}$$

The Dice score is defined as twice the overlapping voxels of the segmentation and ground truth divided by the combined number of voxels of each and shown in equation 3. This value ranges between 0 and 1 and represents the proportion of overlap between the predicted and ground truth segmentations. A value closer to 1 suggests more overlap between the predicted and ground truth segmentations and is therefore preferred. Jaccard Similarity Coefficient is defined as the size of the intersection between the segmentation and ground truth divided by the size of the union of the two and is shown in equation 4. Jaccard Similarity Coefficient, like the Dice Score, ranges from 0 to 1 and explains the similarity between the two sets of segmentation voxels with values closer to 1 being preferred.

$$Dice(A,B) = \frac{2|A \cap B|}{|A| + |B|} \tag{3}$$

$$J(A,B) = \frac{|A \cap B|}{|A \cup B|} \tag{4}$$

Hausdorff Distance is defined as the maximum of the minimum distances between two sets of points (A and B) in space. It is shown in equation 5 for which set A is rewritten as a_i and set B as

b_j. The distance between voxels a_i and b_j is then denoted as δ (a_i, b_j) as the Euclidian Distance between the center of a_i and center of b_j [1]. It describes the distance between two sets of voxels and so a smaller Hausdorff Distance is preferred. Because of the nature of the equation, Hausdorff Distance becomes very sensitive to noise [1]. This issue can be addressed using a Modified Hausdorff Distance which replaces the maximum distance with average distance [2]. The equation for Modified Hausdorff Distance can be seen in equation 6.

$$HD(A,B) = \max\left\{max_i\, min_j\, \delta\left(a_i, b_j\right), max_j\, min_i\, \delta\left(a_i, b_j\right)\right\} \quad (5)$$

$$MHD(A,B) = \frac{1}{|A|}\sum{}_i min_j\, \delta\left(a_i, b_j\right) + \frac{1}{|B|}\sum{}_j min_i\, \delta\left(a_i, b_j\right) \quad (6)$$

The results of the classification model were evaluated using the sensitivity, specificity, PPV, F-Score, and area under the receiver operating characteristic curve (AUC). F-score ranges from 0 to 1 and is calculated from the precision (also called PPV) and recall (also called sensitivity) as their harmonic mean [3]. The equation for F-score can be seen in Equation 7. The AUC represents the probability of the classifier to rank a randomly chosen positive instance higher than a randomly chosen negative instance [4]. Despite ranging from 0 to 1, a perfectly random guessing results in a diagonal line with an AUC of 0.5 and so the more realistic range for an AUC is instead from 0.5 to 1.

$$FScore = \frac{2*PPV*Sensitivity}{PPV+Sensitivity} \quad (7)$$

Supplemental Table 1. Summary of the number of training cases used in each active learning model.

Model	Initial Training Cases	Active Learning Cases
Model B	575	0
Model B AL	575	576
Model C	100	0
Model C AL1	100	200
Model C AL2	100	800
Model C AL3	100	1051

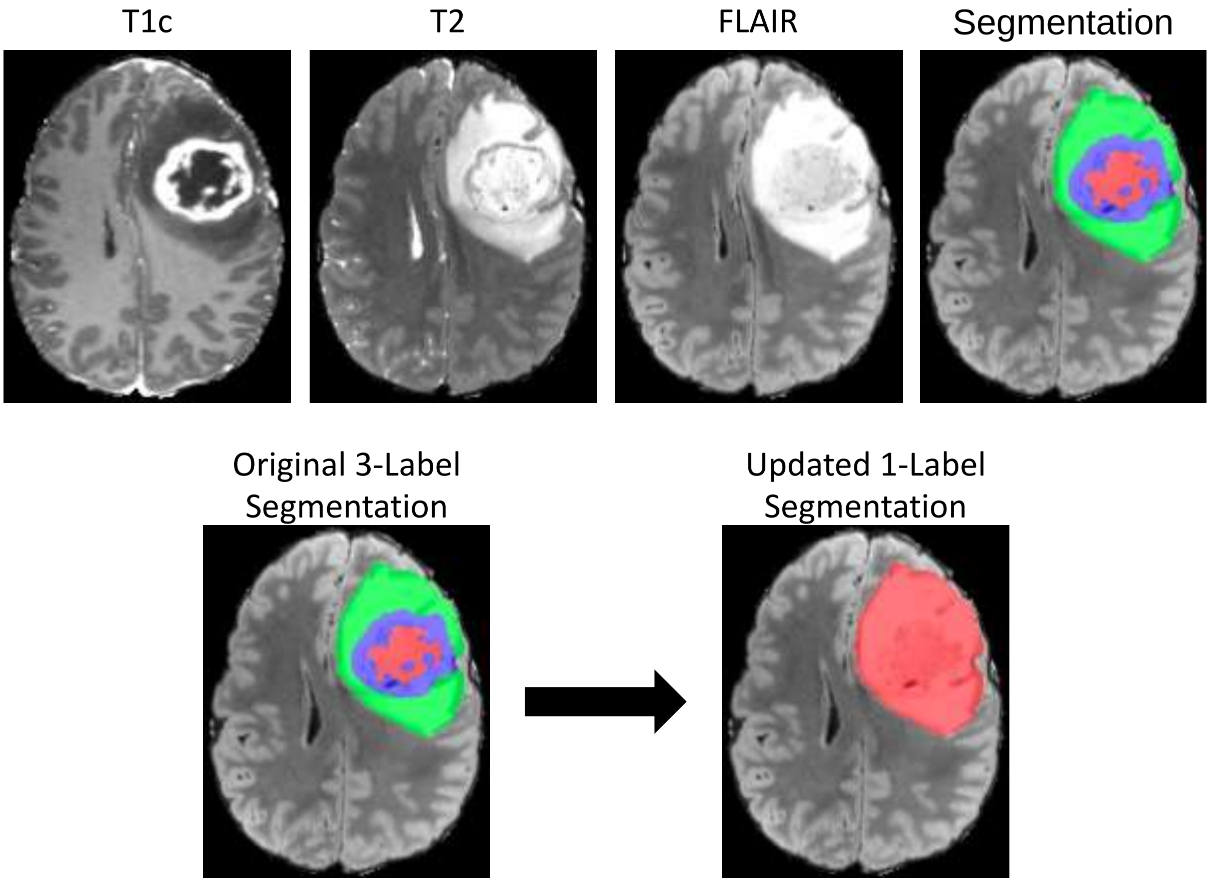

Supplemental Figure 1. A representative case demonstrating (top panel) representative images above from left to right: T1c, T2, FLAIR, and manual ground truth segmentation (red: necrotic core (NCR), blue: Gd-enhancing tumor (ET), green: edematous/invaded tissue (ED)). Bottom panel demonstrates the union of the three segmentation labels for NCR, ET, and ED into a single segmentation label of the whole tumor (WT).

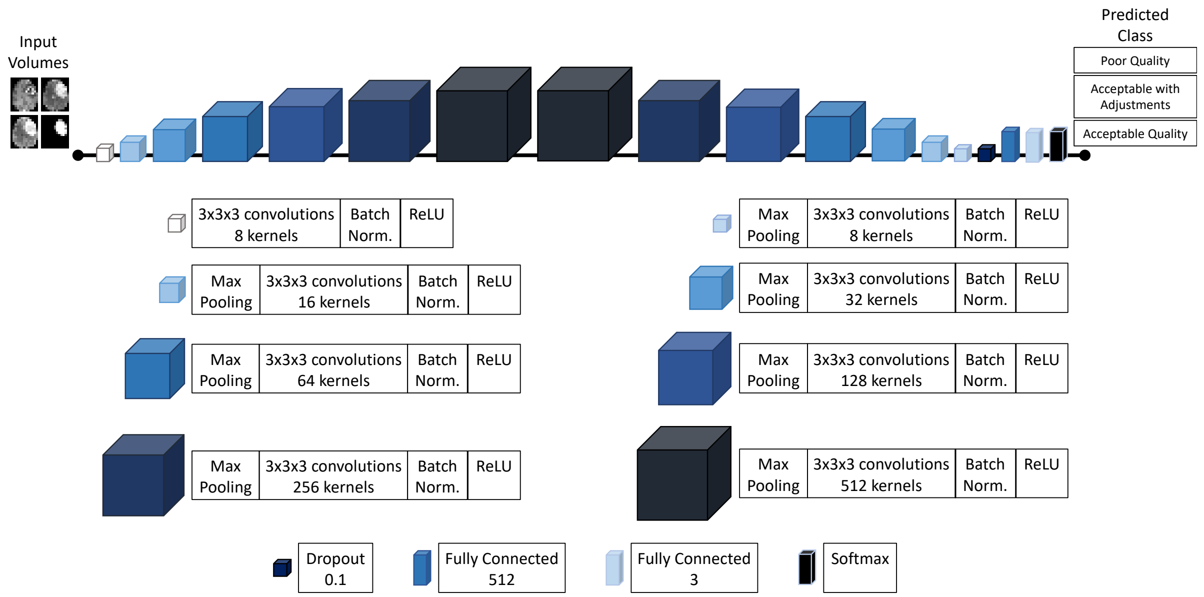

Supplemental Figure 2. Network architecture of the Dice score predictor model for classifying predicted segmentation quality.

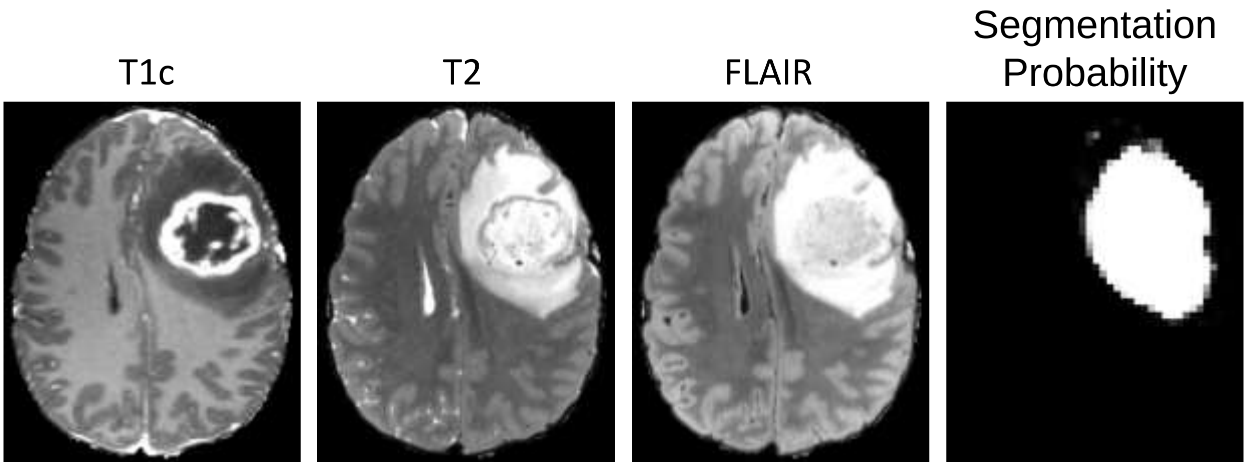

Supplemental Figure 3. Example input images for the Dice score predictor including from left to right: T1c, T2, FLAIR, and the predicted segmentation probability map.

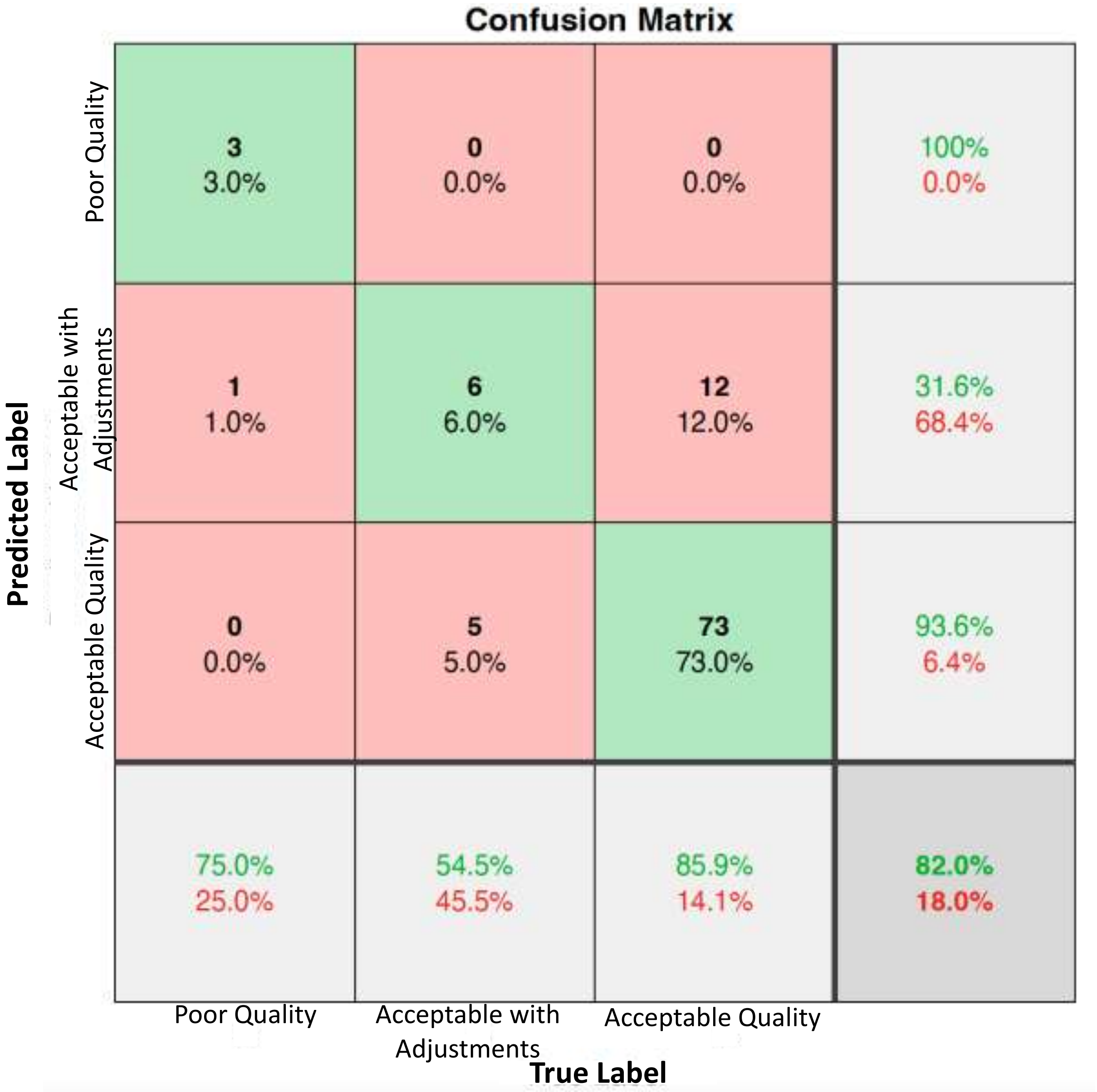

Supplementary Figure 4. Confusion matrix for the classification of predicted segmentations into "Poor Quality", "Acceptable with Adjustments", and "Acceptable Quality".

Discussion

In this study, the application of an active learning approach to segment whole brain gliomas from MRI was assessed. The key benefit to the active learning concept lies in its potential reduction of data requirements, with preferential data being selected for model training through feedback from the model. After three baseline segmentation models were trained as reference, active learning was applied to the two models of reduced dataset size using a Dice score threshold and the training sets were updated based on the queried data. While this first step allowed for the assessment of the viability of active learning in training glioma segmentation models as a concept, it relied on prior knowledge of the ground truth data for the unseen cases to compute Dice scores. In a clinical or real-world setting, this would not be practical as one would want to utilize all available training data that is accompanied by a ground truth segmentation to train the best model possible. A secondary Dice score predictor was then developed to address this challenge with the goal of classifying predicted segmentations into those of "Poor Quality", "Acceptable with Adjustments", and "Acceptable Quality" using Dice score thresholds of below 0.6, between 0.6 and 0.8, and above 0.8, respectively.

Because the use of the Dice score in selecting cases for active learning is not feasible in a real world setting with a lack of ground truth data, it was also important to demonstrate that the classification model could be applied to the active learning itself more than just as a concept. For this evaluation it was compared with the results of a model using the Dice scores. In both models, a drastic reduction in the number of cases requiring manual ground truth segmentations was seen, with the reference Dice method model requiring just 46% of the ground truth images and the classification method model requiring just 43%. The similarity in the reduction of ground truth cases was also mirrored by a similarity in segmentation performance with the Dice method model demonstrating a Dice score performance of 0.885 and the classification method model demonstrating a Dice score performance of 0.860. Comparable results between the reference method that required manual ground truth data and the Dice score predictor method that was more representative of a real-world situation demonstrated that an active learning approach can be a viable technique when facing real world situations rather than just in proof-of-concept settings.

The results of quantitative analysis of the segmentation models demonstrated that an active learning approach when applied to glioma segmentation from MR images shows comparable segmentation results to reference non-active learning models but at a lower ground truth cost. With active learning, the average Dice score of the predicted segmentations of T100 rose from 0.865 to 0.870 for Model B and from 0.825 to 0.868 for Model C. While these two models did not quite reach the Dice score of the reference Model A (0.906), the Dice scores were still comparably high and with much less manual segmentation required for training. For Model B, only 127 of the additional 576 cases required manual segmentation for a total of 702 of the 1151 cases. This reduced the total number of cases needing an expert's manual segmentation by 449 or 39.0% of the total training dataset. For Model C, across all 3 rounds of active learning only 229 of the additional 1051 cases required manual segmentation, reducing the number of total training cases with expert manual segmentation by 822 and meaning that only 329 or 28.6% of the 1151 cases

required manual segmentation. These drastic reductions in manual segmentation required would greatly save in the cost of time and labor by trained experts. Though the segmentations through active learning did not quite reach the levels of the reference model, there is a trade-off in which the reductions of manually segmented ground truth data required can make up for this. This may be especially useful in tasks for which there is more leniency in the precision and so the slight decrease in accuracy of the predicted segmentations is less important compared with the time and effort saved.

Though not for glioma segmentation specifically, various other studies have also implemented active learning techniques to medical image segmentation toward reducing manual segmentation data requirements. In a study applying active learning to interactive 3D image segmentation [5], an active learning technique involving uncertainty fields based on boundary, regional, smoothness and entropy terms was applied and tested on various segmentation tasks including putamen from brain MRI, liver in abdominal CT, and pelvic bones and muscles in both CT and MRI. The study found that in addition to either comparable or improved Dice scores, the active learning techniques also reduced user input by an average of 64%. This finding shows a similar reduction in human effort of segmentation as the present study with a 61% reduction in Model B and a 72.4% reduction in Model C. Another study focusing on generation of realistic chest x-ray images using a conditional generative adversarial network followed by a Bayesian neural network to calculate informativeness for active learning [6] similarly found that an active learning framework was able to achieve comparable results using only 35% of the full dataset. In a study of hippocampal segmentation from MR images [7], a Query-by-Committee approach to active learning was implemented and was able to achieve full segmentation accuracy using only 23% of the dataset. While these studies all show drastic reductions in data requirements, they each also use different approaches to the application of active learning concepts. This suggests two things— first, there are many different techniques to approach active learning while achieving similarly small data requirement results; second, with multiple possible techniques there may be an approach that works best for a given task and so future studies wishing to optimize the process may need to test multiple approaches.

References

[1] Hatt M, Lee JA, Schmidtlein CR, Naqa IE, Caldwell C, De Bernardi E, et al. Classification and evaluation strategies of auto-segmentation approaches for PET: Report of AAPM task group No. 211. Med Phys 2017;44(6):e1-e42.

[2] Aspert N, Santa-Cruz D, Ebrahimi T. MESH: measuring errors between surfaces using the Hausdorff distance. Proceedings IEEE International Conference on Multimedia and Expo 2002;1:705-8.

[3] Taha AA, Hanbury A. Metrics for evaluating 3D medical image segmentation: analysis, selection, and tool. BMC Med Imaging 2015;15:29.

[4] Fawcett T. ROC Graphs: Notes and Practical Considerations for Researchers. Machine Learning 2004;31:1-38.

[5] Top A, Hamarneh G, Abugharbieh R. Active learning for interactive 3D image segmentation. Med Image Comput Comput Assist Interv 2011;14(Pt 3):603-10.
[6] Mahapatra D, Bozorgtabar B, Thiran JP, Reyes M. Efficient Active Learning for Image Classification and Segmentation using a Sample Selection and Conditional Generative Adversarial Network. arXiv 2018:arXiv: 1806.05473.
[7] Nath V, Yang D, Landman BA, Xu D, Roth HR. Diminishing Uncertainty Within the Training Pool: Active Learning for Medical Image Segmentation. IEEE Trans Med Imaging 2021;40(10):2534-47.
